# A survey examining the relationship between burnout, professional empowerment, and personality traits of midwives of an inner London NHS Trust

**DOI:** 10.18332/ejm/184208

**Published:** 2024-04-03

**Authors:** Juan Soria, Karyofyllis Zervoulis, Angeliki Bolou

**Affiliations:** 1 The Royal London Hospital, Barts Health NHS Trust, Maternity Department, London, United Kingdom; 2 London Metropolitan University, London, United Kingdom; 3 School of Health Sciences, Institute for Lifecourse Development, Centre for Chronic Illness and Ageing, Faculty of Education, Health and Human Sciences, University of Greenwich, London, United Kingdom

**Keywords:** burnout, empowerment, personality traits, midwifery, attrition, mental health

## Abstract

**INTRODUCTION:**

Besides the well-known negative effects on physical and psychological well-being, burnout has been associated with high attrition and absenteeism in the midwifery profession. This study explores whether burnout in midwifery can be explained by the midwives’ type of personality and the sense of empowerment they experience at work. Moreover, the study identifies areas of improvement in relation to these topics and elements that can be conducive to strengthening the midwifery workforce.

**METHODS:**

A cross-sectional exploratory study design was used, including an online survey completed by 120 midwives working for an NHS Trust in London. The response rate was 24%. Three validated questionnaires were used: the Copenhagen Burnout Inventory (CBI), the Perception of Empowerment in Midwifery Scale (PEMS), and the Big Five Personality Trait Short Questionnaire (BFPTSQ).

**RESULTS:**

A multiple linear regression analysis indicated empowerment and personality traits are significant predictors of levels of burnout. Furthermore, emotional stability was shown to partially mediate the relationship between empowerment and burnout. The study also examined the midwifery burnout levels of this NHS Trust, which were found to be significantly high and similar to a previous study conducted by the Royal College of Midwives.

**CONCLUSIONS:**

The empowerment experienced by midwives and their personality traits significantly predict the levels of burnout in the midwifery workforce. Only empowerment and emotional stability were significant contributors to the regression model. Multiple strategies can be implemented to support midwives in these two areas. These interventions could also be of great help to reinforce the role of the midwife, making it more appealing to society and, in particular, younger generations with an interest in human-orientated professions.

## INTRODUCTION

Ample evidence has revealed that midwifery work is psychologically demanding. Even when pregnancies and childbirth are straightforward, mothers and families may experience anxiety and rely on midwives for support. This requires midwives to carry a significant amount of emotional work, which often goes unappreciated and predisposes midwives to ‘get on with the job’. When there are challenging working conditions and negative outcomes, this emotional work increases and may leave midwives at risk of secondary trauma. As a result, there are increasing data that indicate a high number of midwives are struggling with burnout, anxiety, and stress^1-4^. This has been strongly associated with high attrition in the midwifery profession in both high- and low-income countries^5,6^.

Schaufeli and Greenglass^7^ defined burnout as ‘a state of physical, emotional and mental exhaustion that results from long-term involvement in work situations that are emotionally demanding’. Although the role of the work environment as a predictor of burnout has been extensively researched, it is crucial to acknowledge the effect that personality types have on the development of this syndrome. A systematic literature review conducted by Angelini^8^ revealed that personality traits were closely related to employees’ burnout risk. The Big Five personality traits model is one of the most widely accepted models to illustrate and measure individual differences in personality. It includes openness to experience, conscientiousness, extraversion, agreeableness, and neuroticism. In recent times, the role of empowerment has become a dominant theme for healthcare workers. Apart from preventing burnout and stress, when staff feel empowered and engaged, they feel more capable of making tangible contributions and are more open to upholding change and improvement, leading to enhanced quality of care^9,10^.

This study proposes to enhance the knowledge around burnout in midwifery with the intention of identifying areas of improvement and elements that can be conducive to strengthening the midwifery workforce. To achieve this, a careful assessment will need to be taken to balance the staff needs with the current challenges that maternity services are experiencing. The first hypothesis explores whether burnout in midwifery can be explained by the sense of empowerment midwives experience at work and their personality traits. A second hypothesis predicts that the relationship between empowerment and burnout will be mediated by personality traits. Moreover, this study also investigates the current midwifery burnout levels in a London NHS Trust according to different demographics such as age, ethnicity, seniority, and practice area.

## METHODS

### Design

A cross-sectional exploratory study design was used to examine the association between the sense of empowerment midwives experience, their personality type, and their levels of burnout.

### Procedure

Participants were recruited by convenience sampling from an NHS Trust in London. The briefing consent form and the link to an online survey were shared via work email, and posters announcing the research were attached in the handover and staff rooms of each maternity unit. Consent to take part in the study was gained by participants clicking the link to the survey. After accessing this link, participants were given a brief description of the study. No identifiable data were collected, and the questionnaires were anonymous. All participants involved took part in the study on a volunteer basis. To prevent missing data, all the questions were made compulsory.

### Ethics

In accordance with the NHS Health Research Authority^11^ guidelines, a Research Ethics Committee review (REC) was not required to conduct this study as this type of work should be managed within the scope of the regular employer/employee relationship in line with routine standards for staff surveys. Despite this, university approval was required and granted by The London Metropolitan University on 6 March 2023. The data collection started on 24 April 2023, and ended on 7 July 2023. This research followed the British Psychological Society Ethics Guidelines for Internet-mediated Research^12^. In order to prevent harm, participants were given the contact details of their NHS Trust ’s wellbeing team and the ‘Samaritans’^13^ organization in case the survey caused them distress.

### Sample

The inclusion criteria involved members of staff registered with the Nursing and Midwifery Council (NMC) and currently practicing as midwives. Approximately 500 midwives were invited to take part in the survey.

### Survey tools

The Perceptions of Empowerment in Midwifery Scale (PEMS) was utilized to evaluate the sense of empowerment midwives experience at work^14^. This scale contains 18 items divided equally across three sub-scales: autonomous practice, effective management, and women-centered practice (in accordance with the terminology used by NHS England, this work uses the terms ‘pregnant woman’ and ‘pregnant women’. It is important to note that this terminology does not intend to exclude any individual who is pregnant or has given birth but does not identify as a woman. Intersex, transgender, and non-binary people who experience pregnancy and childbirth may encounter distinct health inequalities, including limited access and inadequate support in relation to their specific care needs within maternity services). Each subscale has been reported to have a Cronbach’s alpha of 0.7 or more when rounded to one decimal place, which is deemed to be satisfactory^15^. In this study, the overall Cronbach’s alpha is 0.84, which is considered good.

The Big Five Personality Trait Short Questionnaire (BFPTSQ) was utilized to measure the personality traits of the participants. This inventory contains 50 items equally divided among five different categories: openness, conscientiousness, extraversion, agreeableness, and emotional stability (also known as neuroticism)^16^. The Cronbach’s alpha for each subscale in this study was ≥0.7, which is considered satisfactory^15^.

The Copenhagen Burnout Inventory (CBI) was chosen to assess the levels of burnout affecting midwives. This inventory has been used in multiple studies involving midwives. It consists of 19 items divided across three sub-scales: personal burnout, work-related burnout, and client-related burnout^17^. The Cronbach’s alpha for the three subscales has been reported as very high, in the range 0.85–0.87^15^. The Cronbach’s alpha for this survey’s subscales was ≥0.86. All items use a 5-point scale with scores being adapted so that the possible scores for all three areas range from 0 (low burnout) to 100 (severe burnout). A score of 0–50 represents low burnout. A score of 50–74 is considered moderate burnout, while a score of 75–99 represents a high level of burnout. Finally, a score of 100 indicates severe burnout^17^.

### Data analysis

**Figure 1:**
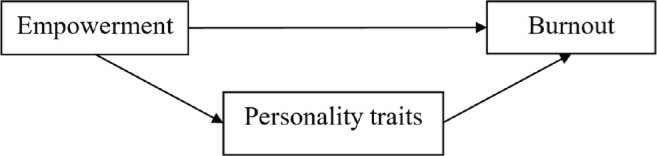
Hypothesised Mediation Analysis model

The data collected was analyzed with the program IBM SPSS Statistics 29. A Pearson correlation coefficient was computed to determine the relationship between the variables of interest: empowerment, personality traits, and levels of burnout. A multiple linear regression analysis was conducted to investigate whether the sense of empowerment that midwives experience at work and their personality traits (independent/predictor variables) could significantly predict levels of midwife burnout (dependent/outcome variable). None of the assumptions for parametric testing was violated. To investigate whether personality traits mediate the relationship between empowerment and burnout, a simple mediation analysis was performed using the Process SPSS macro (Figure 1). A one-way ANOVA test was conducted to determine if there were differences in the overall burnout scores according to age, ethnicity, seniority, and practice area. A Tukey *post hoc* test was used to determine what burnout scores and age groups were significantly different from each other.

## RESULTS

The sample for the current study consisted of 119 females and one non-binary respondent (n=120); 60.8% of the participants were aged 31–50 years, 19.2% were aged 21–30 years, and 20% were aged ≥51 years. The distribution of ethnic groups included White (54.2%), Black (30.8%), Mixed (8.3%), Other (4.2%), Asian (1.7%), and Chinese (0.8%). Of the participants, 29.2% were based in intrapartum areas, 25% were based in the community, 15.8% were specialist midwives, 10% of the respondents were based in antenatal areas, 8.3% were senior midwifery managers, 6.6% were part of the continuity of care teams, and 5% were based on the postnatal ward. The response rate for the online survey was 24%.

The results of the Pearson correlation analysis indicate a significant negative relationship between burnout and empowerment (r[118]= -0.40, p<0.001). There was also a significant negative relationship between burnout and conscientiousness (r[118]= -0.18, p=0.028); burnout and extraversion (r[118]= -0.23, p=0.006); burnout and agreeableness (r[118]= -0.30, p<0.001); and burnout and emotional stability (r[118]= -0.47, p<0.001). There was also a non-significant negative relationship between burnout and openness (r[118]= -0.13, p=0.07) (Table 1).

**Table 1:** Descriptive statistics and correlation coefficients for burnout, empowerment and personality traits of midwives working in an inner-London NHS Trust, England, April –September 2023 (N=120)

	*n*	*Mean*	*SD*	*1*	*2*	*3*	*4*	*5*	*6*	*7*
**1. Burnout**	120	2.98	0.70	-						
**2. Empowerment**	120	3.84	0.48	-0.40**	-					
**3. Openness**	120	3.78	0.55	-0.13	0.09	-				
**4. Conscientiousness**	120	4.20	0.55	-0.18*	0.23**	0.36**	-			
**5. Extraversion**	120	3.74	0.75	-0.23**	0.22**	0.41**	0.48**	-		
**6. Agreeableness**	120	4.20	0.52	-0.30**	0.25**	0.41**	0.60**	0.40**	-	
**7. Emotional stability**	120	3.35	0.82	-0.47**	0.23**	0.21*	0.49**	0.53**	0.45**	-

NHS: National Health Service.

* Pearson correlation significant at p<0.05 level (1-tailed).

** Correlation significant at p<0.01 level (1-tailed).

**Figure 2:**
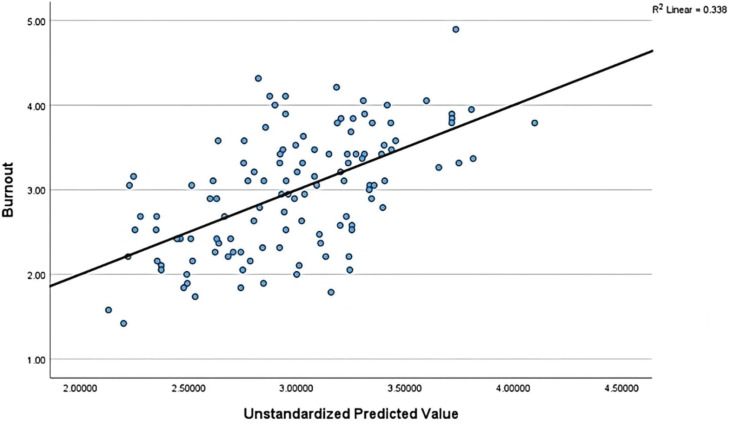
Scatterplot for Regression Model

A multiple linear regression analysis revealed that the association between empowerment, personality traits, and burnout was moderate (r=0.58) (Figure 2). The results of the regression indicated that the model explained 30% of the variance and that the model was a significant predictor of levels of burnout. Empowerment and emotional stability contributed significantly to the model (empowerment, β= -0.32; 95% CI: -0.7 – -0.23, p<0.001, and emotional stability, β= -0.45; 95% CI: -0.56 – -0.22, p<0.001), while openness, extraversion, conscientiousness and agreeableness did not (openness, β= -0.05; 95% CI: -0.29–0.17, p=0.60, extraversion, β=0.07; 95% CI: -0.12–0.25, p=0.51, conscientiousness, β=0.19; 95% CI: -0.02–0.51, p=0.07, and agreeableness, β= -0.14; 95% CI: -0.46–0.09, p=0.18). These results confirmed the first part of the first hypothesis, namely, that if empowerment increases by one unit, burnout will decrease by 0.47; however, the second part of the first hypothesis was only supported in relation to emotional stability. If emotional stability increases by one unit, burnout will decrease by 0.39 (Table 2).

**Table 2:** Multiple linear regression analysis with dependent variable burnout and independent variables empowerment and personality traits. The sample included midwives working in an inner-London NHS Trust, England, April – September 2023 (N=120)

*Independent variables*	*B*	*SE*	*β*	*t*	*Sig.*	*95% CI for β*	
						*Lower*	*Upper*
Empowerment	-0.47	0.12	-0.32	-3.95	<0.001	-0.70	-0.23
Openness	-0.06	0.11	-0.05	-0.53	0.60	-0.29	0.17
Extraversion	0.06	0.09	0.07	0.66	0.51	-0.12	0.25
Agreeableness	-0.19	0.14	-0.14	-1.34	0.18	-0.46	0.09
Conscientiousness	0.24	0.13	0.19	1.80	0.07	-0.02	0.51
Emotional stability	-0.39	0.08	-0.45	-4.61	<0.001	-0.56	-0.22

B: unstandardized coefficient. β: standardized coefficient.

The results from the mediation analysis revealed that only emotional stability had a significant indirect effect on levels of burnout (b= -0.15, t= -2.15). The direct effect of empowerment on burnout in the presence of the mediator was also significant (b= -0.47, p<0.001). Hence, emotional stability partially mediated the relationship between empowerment and burnout (Table 3).

**Table 3:** Mediation analysis summary for burnout, empowerment (EM) and emotional stability (ES) of midwives working in an inner-London NHS Trust, England, April – September 2023 (N=120)

*Relationship to burnout*	*Total effect*	*Direct effect*	*Indirect effect*	*Confidence interval*		*t-statistic*	*Conclusion*
				Lower bound	Upper bound		
**ES**			-0.15	-0.31	-0.04	-2.15	
**EM in presence of ES**		-0.47					Partial mediation by ES
**EM + ES**	-0.62	-0.47	-0.15				

### Burnout levels

In relation to the current burnout levels for the midwifery staff at this NHS Trust, the results determined that midwives’ levels of personal and work-related burnout were moderate to high (Table 4). The mean score for personal burnout was 69.78, while the work-related mean score was 66.12 (a mean score >50 is considered to signify burnout). In this study, 50.8% of midwives (n=61) reported moderate levels of personal burnout (scores 50–74), while another 40% (n=48) reported high levels (scores 75–99). There were 1.7% (n=2) of midwives who scored 100. In total, 93% (n=111) of midwives scored moderate to severe on the personal burnout domain. In terms of work-related burnout, 37.5% (n=45) of midwives recorded moderate levels, while 39.2% (n=47) had high levels, and 0.8% (n=1) had severe work-related burnout. In total, 77.5% (n=93) of midwives registered moderate to severe levels of work-related burnout. In contrast, client-related burnout was relatively low, with a mean score of 41.74. Of the 120 midwives completing this scale, 72.5% (n=87) had a score of <50. Of the remaining participants, 20% (n=24) had moderate levels of client burnout, 5.8% (n=7) had high levels, and <2% were observed to have severe client-related burnout (n=2; 1.7%).

**Table 4:** Prevalence of burnout scores, and reliability of the Copenhagen Burnout Inventory for each subcategory of this inventory, among midwives working in an inner-London NHS Trust, England, April – September 2023 (N=120)

*Scores*	*n (%)*	*Cronbach alpha*
**Overall**		0.92
No/Low (<50)	35 (29.2)	
Moderate (50–74)	63 (52.5)	
High (75–99)	22 (18.3)	
Severe (100)	0 (0.0)	
Mean (SD)	59.58 (14.06)	
**Personal**		0.90
No/Low (<50)	9 (7.5)	
Moderate (50–74)	61 (50.8)	
High (75–99)	48 (40.0)	
Severe (100)	2 (1.7)	
Mean (SD)	69.78 (15.76)	
**Work**		0.86
No/Low (<50)	27 (22.5)	
Moderate (50–74)	45 (37.5)	
High (75–99)	47 (39.2)	
Severe (100)	1 (0.8)	
Mean (SD)	66.12 (16.51)	
**Client**		0.87
No/Low (<50)	87 (72.5)	
Moderate (50–74)	24 (20.0)	
High (75–99)	7 (5.8)	
Severe (100)	2 (1.7)	
Mean (SD)	41.74 (18.23)	

A one-way ANOVA test showed that only the differences in scores according to age groups were statistically significant: ANOVA (F [4.115]=6.47, p<0.001). The means and standard deviations are presented in Table 5. All the assumptions for parametric testing were met. A Tukey *post hoc* test revealed statistically significant differences in the mean burnout scores between those: aged ≥61 years and 22–30 years (p<0.001); aged ≥61 and 31–40 years (p=0.002); aged ≥61 and 41–50 years (p=0.01); and aged 51–60 and 22–30 years (p=0.01). There was no statistically significant difference between the other group combinations with burnout: ethnicity, seniority, and practice area.

**Table 5:** Burnout scores by age groups for midwives working in an inner-London NHS Trust, England, April – September 2023 (N=120)

*Age (years)*	*n*	*Mean*	*SD*
22–30	23	3.31	0.64
31–40	40	3.10	0.63
41–50	33	3.00	0.67
51–60	16	2.62	0.67
≥61	8	2.13	0.60
Total	120	3.00	0.70

## DISCUSSION

This study explored the association between empowerment, personality traits, and burnout for midwives working in an NHS Trust in London. The analysis revealed that empowerment and personality traits significantly predict levels of burnout in the midwifery workforce. Nonetheless, only empowerment and emotional stability were significant contributors to the regression model. The higher these two elements are, the lower the levels of burnout. The Pearson correlation results showed that as the levels of empowerment, conscientiousness, extraversion, agreeableness, and emotional stability increase, the levels of burnout decrease.

In addition, emotional stability was found to partially mediate the relationship between empowerment and burnout.

These results suggest that empowering midwives and supporting their emotional well-being can be an effective strategy to decrease burnout levels. These findings are supported by numerous studies conducted across the healthcare sector^9,10,17,18^. Decreasing job burnout has the potential to improve employees’ well-being, boost job performance, and reduce public health costs due to turnover and absenteeism^8^. Moreover, evidence demonstrates that fostering a culture that promotes a sense of autonomy, belonging, and contribution can transform the working environment, enabling increased efficiency and enhanced patient safety^19^.

Maurer^20^ described six basic items as the foundation of effective empowerment efforts. He argued that staff want basic things from their work: meaning, results, challenges and learning opportunities, respect and recognition, autonomy, and affiliation (being part of a bigger team). These aspects of empowerment are directly and indirectly addressed in the recently published ‘Three Year Delivery Plan for Maternity and Neonatal Services’ by NHS England^21^. There are four main themes in the report.

The first theme includes the promotion of the continuity of care model. This system has been proven to not only prevent burnout but also improve health outcomes, decrease costs, and increase satisfaction for both service users and staff^4,22^.

The second theme looks into the workforce, which is one of the biggest modifiable risk factors for burnout. One of the specific objectives in this area is to ensure there are adequate staffing levels to optimize the care for women and babies. In relation to staff recognition, one of the goals is to make sure staff feel valued and have a rewarding and sustainable career within the NHS. This includes investing in the staff to ensure they have ongoing training and career development opportunities, facilitating flexible working hours, and providing support when employees are approaching retirement age to permit them to continue to use their skills and experience. Part of the workforce agenda for 2023–2024 involves the provision of funding for every maternity unit to recruit a retention midwife^19^. Another important element on the NHS England agenda, was the introduction of the ‘well-being lead’ roles; the NHS Health and Well-being Framework was first published in 2018 and is widely used by many NHS organisations^23^. In relation to promoting autonomy, giving staff control over decisions about rotation around different clinical areas may prevent staff from leaving for other jobs or retiring early^24^.

The third theme from the Three-year Delivery Plan discusses the need to develop and sustain a culture of safety, learning, and support. NHS England aims to ensure staff are supported with professionalism, kindness, compassion, and respect. Part of this transformation also includes tackling incivility. Apart from increasing safety, civility is also crucial to promote staff well-being and increase productivity^25^. In view of this, Higher Education England^26^ developed a ‘Civility toolkit’ to support organizations ‘growing a culture of kindness’. There is also a big focus on the need to train as a multidisciplinary team, ‘Staff who work together must train together’^27^.

The fourth theme relates to structures and standards that reinforce safer, more personalized, and more equitable outcomes. One of the key messages in this area is the commitment to ensure all healthcare professionals are supported in making optimal use of digital resources with satisfactory computer hardware, optimized Wi-Fi, secure networks, and training^19^.

Promoting the emotional well-being of individual midwives is becoming particularly accepted as an essential approach in recruiting, retaining staff, and sustaining a healthy midwifery workforce^19^. The latest figures show the attrition levels for the midwifery profession continue to increase by the month. The current shortage of about 2677 midwives in England leaves the NHS incapacitated to deliver high-quality care for women, babies, and families^28^.

A different strategy implemented by NHS England to enhance the support midwives receive has been the introduction of the Professional Midwifery Advocate (PMA) role in order to deploy the ‘Advocating for Education and Quality Improvement’ (A-EQUIP) model. The implementation of the A-EQUIP started in 2017, and it aims to facilitate a continuous improvement process that values midwives, builds their personal and professional resilience, and contributes to the provision of high-quality care^29^.

With regard to emotional stability, the results confirming a positive association between neuroticism and midwifery burnout constitute an important addition to the existing knowledge. These results also mirror other studies, including different professionals working within people-oriented professions, such as policemen or school teachers^8^. A study conducted by Bianchi^30^ revealed that neuroticism explained more variance in burnout than work-related factors, including professional support.

An innovative venue to decrease burnout by lowering neuroticism is mindfulness meditation. Research around neuroticism has shown that mindfulness is a powerful element to consider in relation to improving emotional stability. Mindfulness nurtures several positive psychological effects, including improved subjective well-being, decreased mental health symptoms and emotional reactivity, and enhanced behavioral regulation^31^.

The burnout levels reported in this study revealed significant emotional distress by the majority of participants. Although the client-related burnout scores were relatively low, with only 27.5% of the participants scoring moderate to severe, most of the midwives (93%) scored moderate to severe on the personal burnout domain, and 77.5% recorded moderate and above scores for work-related burnout. These high levels of emotional distress are not a surprise; in fact, they are similar to the UK Work, Health and Emotional Lives of Midwives (WHELM) study commissioned by the Royal College of Midwives^32^, which revealed the poor emotional well-being of UK midwives in terms of stress levels, anxiety, depression, and burnout. The WHELM study reported that 83% of the midwives scored moderate and above for personal burnout, and 67% recorded moderate and above for work-related burnout. Client-related burnout was low at 15.5%. The levels of burnout reported in the present study are significantly higher compared to population averages and other WHELM collaborating countries. For example, looking at the Australian arm, 65% scored high for the personal domain, 43.8% scored high for the work-related domain, and 10.4% scored high for the client-related category. In the Swedish arm, 39.5% scored high in personal burnout, while 15% scored high in the work-related and client-related areas. In the Norwegian arm, 20% of the midwives scored high on personal burnout, the same percentage on work-related burnout, and only 5% scored high in the client-related category^1,32^. The comparatively low client-related burnout scores are worth pondering over. These results suggest that most of the midwives do not find it difficult to work with women. Burnout was much more likely to be associated with work discontentment and personal factors. The relevance of the midwife–woman relationship to midwives has been recognized in several studies, and it would be interesting to investigate whether this has a protective function in terms of burnout reduction^32^. A summary of the strategies discussed is presented in Table 6, which includes the potential effect on empowerment and emotional stability.

**Table 6:** Summary of the strategies to decrease burnout by supporting emotional stability (ES) and empowerment (EM) of midwives. Study conducted in midwives working in an inner-London NHS Trust, England, April – September 2023 (N=120)

*Individualized support*	*Organizational elements*
Mindfulness training - ↑ES	Staffing levels/skills mix - ↑ES ↑EM
Promoting autonomy - ↑EM	Continuity of carer model - ↑ES ↑EM
Pastoral support (PMA role) - ↑ES ↑EM	Multidisciplinary training - ↑ES ↑EM
Career developing opportunities - ↑EM	Optimized digital resources - ↑EM
Staff recognition - ↑EM	Promoting civility - ↑ES ↑EM
Flexible working hours - ↑EM	Retention midwife - ↑ES ↑EM
	Well-being leads - ↑ES

Similar to multiple international studies, this study identified that younger, recently qualified midwives tend to report higher levels of burnout compared to older midwives^23,33^. Doherty and O’Brien^33^ explored whether young midwives are better prepared to engage in efficient self-care strategies compared to older midwives. It was argued that nowadays, there is more awareness of the importance of self-care; nonetheless, it was also suggested that young people have unrealistic expectations of the world while having a diminished ability to cope. Moreover, it was highlighted that the concerns articulated about young midwives reflect societal changes, meaning that it is not just younger midwives who have less resilience than their senior colleagues, but that individuals from new generations, at large, are not as resilient as people from older generations.

### Strengths and limitations

The research around burnout, empowerment, and personality traits in midwifery is limited. This study constitutes a cornerstone that may prompt further investigations into this subject. In order to advance the knowledge in this area, longitudinal studies are needed to better understand the catalysts for burnout. An interesting angle to consider is whether burnout could be the main cause affecting emotional stability rather than the other way around. This could be studied with newly graduated midwives, which would allow them to establish a baseline for their emotional stability level and observe how this will change over the years of midwifery practice. This would facilitate the development of targeted strategies to improve emotional stability and prevent and neutralize burnout, which ultimately would contribute to improving midwifery retention. This research used three validated established tools, which increases the validity of the results.

The sampling method used in the study, convenience sampling, is susceptible to self-selection bias. This type of bias appears when people are permitted to decide whether they want to participate in a research study. Since participants often deviate from non-participants in ways meaningful to the research, self-selection can lead to a biased sample and affect the generalizability of the results. Although this study provides a helpful snapshot on this subject, it does not claim representativeness. However, the sample included multi-ethnic midwives working across different settings within an inner-city NHS Trust, and the response rate (24%), although relatively low, is above average for similar studies conducted around this topic, which increases the study’s validity. Picker^34^ suggested it would be wrong to generalize what constitutes an acceptable survey response rate. High response rates are desirable because they are usually linked to highly representative data; however, this is not always the case. Surveys with lower response rates can be highly representative. It should be noted that the presence of high burnout levels in the work environment may have influenced other midwives’ willingness to participate in the present study.

## CONCLUSIONS

The results of this study provide insight into the relationship between empowerment, personality traits, and burnout. The burnout syndrome is multifaceted and subjective, so it can be difficult to understand and prevent its development. Empowering midwives and supporting their emotional stability were found to be the most relevant elements in relation to preventing and neutralizing burnout. Introducing mindfulness training as part of the mandatory educational training for midwives may constitute an interesting venue to consider in order to promote emotional stability.

The different strategies presented in this study have the potential to strengthen the midwifery workforce by empowering midwives and supporting their emotional stability, which will reduce burnout and contribute to improving midwifery retention and decreasing sickness rates. Moreover, these interventions can be of great help to make the role of the midwife more attractive and appealing for our society and, in particular, younger generations with an interest in human-oriented professions.

## Data Availability

The data supporting this research are available from the authors on reasonable request.
